# Asymptomatic Plantar Nodules in a Toddler

**DOI:** 10.7759/cureus.30611

**Published:** 2022-10-23

**Authors:** Carli P Whittington, Simran Kalsi, Katelyn E Shea, Keith W Morley

**Affiliations:** 1 Department of Medicine, Division of Dermatology, University of Vermont Medical Center, Burlington, USA; 2 Department of Medicine, Division of Dermatology, The Robert Larner College of Medicine, University of Vermont Medical Center, Burlington, USA

**Keywords:** precalcaneal congenital fibrolipomatous hamartoma, nodule, benign, pediatric, heel, foot, plantar

## Abstract

Precalcaneal congenital fibrolipomatous hamartoma (PCFH) is a rare benign skin lesion that typically presents at birth, or within the first several years of life, as single or multiple asymptomatic skin-colored papules or nodules on the plantar heels. We present a classic case of PCFH in a 3-year-old child. This uncommon entity has no reported malignant features or malignant transformations. We demonstrate how this diagnosis can be made clinically without subjecting pediatric patients to potentially painful, traumatizing, costly skin biopsies and unnecessary imaging.

## Introduction

Precalcaneal congenital fibrolipomatous hamartomas (PCFH), also known as bilateral congenital adipose plantar nodules, are uncommon benign neoplasms that develop on the plantar feet at birth or within the first few years of life [1]. They may be unilateral or bilateral, typically presenting with normal overlying skin and no associated symptoms. PCFHs are hypothesized to arise from incomplete regression of fetal tissue, fat herniation through defects in the plantar fascia, or underlying genetic mechanisms [2]. The lesions have been reported to spontaneously resolve between two to three years of age and are not associated with malignant transformation [3]. We present an uncommon case of a three-year-old child with asymptomatic bilateral nodules on the medial aspect of the plantar feet, diagnosed clinically as PCFH. We demonstrate that PCFH can be diagnosed without subjecting young patients to potentially traumatizing, painful, and costly skin biopsy or unnecessary imaging.

## Case presentation

**Figure 1:**
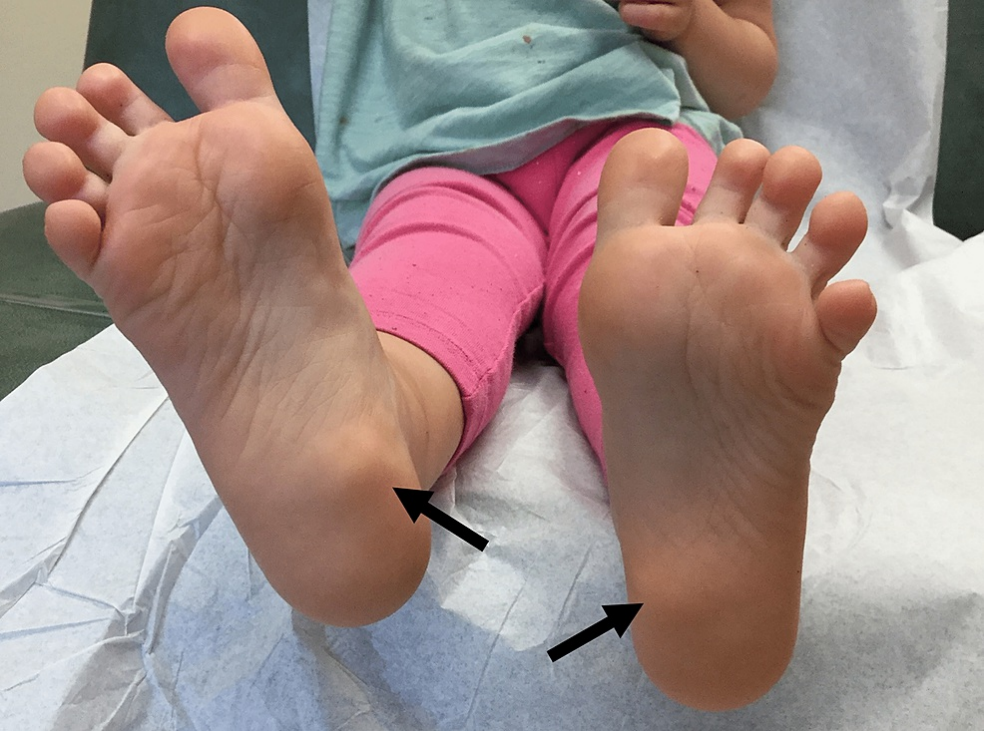
Skin-colored, rubbery subcutaneous nodules on the bilateral plantar feet.

**Figure 2:**
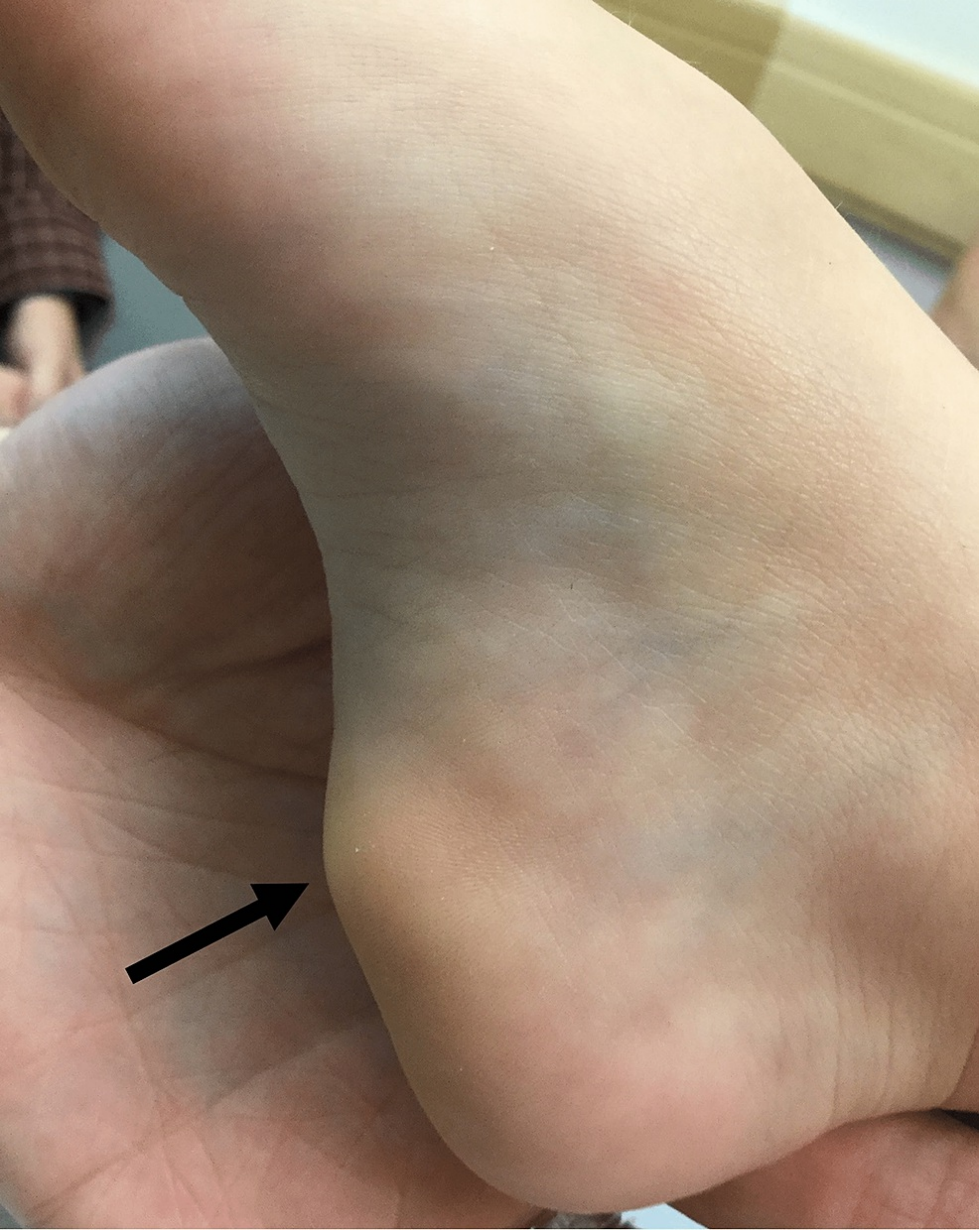
Subcutaneous nodule involving the medial plantar foot.

A healthy three-year-old female presented with rubbery nodules on the bilateral plantar feet, present since birth. There was no family history of similar lesions. The lesions grew slowly and proportionately with age. Parents denied changes in the patient’s gait or perceived pain related to the nodules. Physical examination revealed two soft, mobile, non-tender, skin-colored subcutaneous nodules 1.5 cm and 2.0 cm in diameter overlying the bilateral plantar calcanei (Figures 1, 2). No bleeding, purulent drainage, or overlying skin changes were noted. The patient was clinically diagnosed with precalcaneal congenital fibrolipomatous hamartoma (PCFH) and the parents were reassured accordingly.

## Discussion

PCFH is a benign neoplastic process that typically presents at birth or within the first several years of life as one or more asymptomatic, skin-colored papules or nodules on one or both plantar heels [1]. Lesions may remain stable in size or grow in proportion to patient growth. PCFH is not associated with any reported malignant features or malignant transformation. While the etiology of PCFH is unclear, hypotheses include incomplete regression of fetal tissue, defects in the plantar fascia, or genetic mutation [2,3]. While rare and sporadic in occurrence, some authors suggest that PCFH may be underreported and may have a familial component [4-7]. In most cases, such as with our patient, PCFH can be diagnosed clinically [8]. Skin biopsy and/or ultrasonography may be pursued in cases of diagnostic uncertainty. Differential diagnoses include piezogenic pedal papules, juvenile plantar fibromatosis, calcified nodules, nevus lipomatosus superficialis, focal dermal hypoplasia, congenital solitary histiocytoma, and congenital hemangioma.

If biopsied, PCFH histology is characterized by dermal lobules of mature adipocytes surrounded by collagenous fibrous sheaths without overlying epidermal changes [2]. Unfortunately, a skin biopsy can be traumatic for pediatric patients, potentially leading to a lasting aversion to doctor visits as well as significant post-procedural pain and discomfort while walking given that the plantar surface is involved. If imaged, ultrasonography reveals hypoechoic bands intertwined with a homogenous, hyperechoic subcutaneous mass representing adipose tissue surrounded by collagen fibers [3,8]. X-ray, computed tomography, or magnetic resonance imaging can be used but may be costly, time-consuming, and expose patients to significant amounts of radiation [9]. PCFH treatment predominantly involves parental reassurance, as there are reports of spontaneous resolution by two to three years of age. PCFH does not usually require invasive interventions, although foot orthotics may help. If affecting gait, symptomatic, or anatomically disfiguring, the nodules can be surgically excised [10].

## Conclusions

PCFHs are uncommon, benign, and typically asymptomatic lesions that can develop from birth to early childhood and may spontaneously resolve by two to three years of age. In this case of PCFH in a three-year-old, the patient’s parents were reassured regarding the nature of PCFH and no further work-up or treatment was recommended. Follow-up one year later revealed asymptomatic, stable-sized lesions that did not bother the patient, further supporting the diagnosis. This report demonstrates that classic clinical features can be used to make this rare and likely underrecognized diagnosis without subjecting young patients to avoidable and potentially traumatic and costly lesion biopsy and unnecessary imaging.
